# Anthropometric Improvement among HIV Infected Pre-School Children Following Initiation of First Line Anti-Retroviral Therapy: Implications for Follow Up

**DOI:** 10.1371/journal.pone.0167565

**Published:** 2016-12-28

**Authors:** Atnafu Mekonnen Tekleab, Birkneh Tilahun Tadesse, Ababi Zergaw Giref, Damte Shimelis, Meseret Gebre

**Affiliations:** 1 St Paul’s Hospital Millennium Medical College, Addis Ababa, Ethiopia; 2 Hawassa University, Department of Pediatrics, Hawassa, Ethiopia; 3 Addis Ababa University, School of Medicine, Addis Ababa, Ethiopia; 4 ALERT Medical Center, Addis Ababa, Ethiopia; UNAIDS, GUYANA

## Abstract

**Background:**

Antiretroviral therapy (ART) is a lifesaving intervention for HIV infected children. There is a scarcity of data on immunological recovery and its relation with growth indicators among HIV infected young children. The current study aims to assess the pattern of anthropometric Z-score improvement following initiation of first-line ART among under-five children and the relationship between anthropometric Z-score improvement and immunologic recovery.

**Methods:**

We included under-five children who were on first-line ART at five major hospitals in Addis Ababa, Ethiopia. We measured anthropometry and collected clinical and laboratory data at follow up, and we retrieved clinical and anthropometric data at ART initiation from records. Z-scores for each of the anthropometric indices were calculated based on WHO growth standards using ENA for SMART 2011 software. Linear regression was used to assess the relationship between time on ART and anthropometric Z-score improvement; and the relationship between anthropometric Z-score improvement and immunologic recovery. Multiple linear regression was used to assess the independent predictors of anthropometric Z-score change.

**Results:**

The median age of the participants was 4.1 (Interquartile range (IQR): 3.3–4.9) years. More than half (52.48%) were female. The median duration of follow up was 1.69 (IQR: 1.08–2.63) years. There was a significant improvement in all anthropometric indices at any follow up after initiation of first-line ART (underweight; 39.5% vs16.5%, stunting; 71.3% vs 62.9% and wasting; 16.3% vs 1.0%; p-value< 0.0001). There was an inverse relationship between improvement in weight for age Z-score (WAZ) and duration of ART (R^2^ = 0.04; F (1, 158); p = 0.013). Height for age Z-score (HAZ) both at the time of ART initiation and follow up has a positive linear relationship with CD4 percentage at follow up (Coef. = 1.92; R^2^ = 0.05; p-value = 0.002). Duration on ART (*Std*. *Err*. = 0.206, *t* = -1.99, p-value = 0.049) and level of maternal education (*Std*. *Err*. = 0.290, *t* = 2.64, p-value = 0.009) were the only independent predictors of the change in WAZ and change in HAZ at any follow up visit respectively.

**Conclusion:**

There was a significant improvement in all anthropometric indices at any follow-up after initiation of first-line ART among under-five children. HAZ was linearly related with immunologic recovery following ART initiation. The findings indicate that anthropometric indices could be taken as proxy indicators of immunologic recovery for under-five children.

## Introduction

Malnutrition and infection are closely related [1]. HIV infection increases the risk of malnutrition through different mechanisms including loss of appetite and disturbed intestinal function [1–3]. Metabolic changes and abnormal cytokine production also contribute for the occurrence of malnutrition in HIV patients [3]. It is well established that the calorie requirement of HIV infected people is higher than their uninfected counter parts [3, 4]. Furthermore, the amount of extra calorie needed by the HIV infected child depends on whether the child is symptomatic or not; a higher calorie is needed by those who are severely ill [5].

Different studies explored the relationship between malnutrition and HIV infection. HIV infected children were found to be more wasted, more underweight and tended to be more stunted than HIV negative malnourished children [6, 7]. The significant effect of HIV as a cause of malnutrition in children was also reported by Saloojee *et a*l [8]. Non edematous severe malnutrition was reported to be more common in HIV infected children as compared to the edematous type [9, 10]. The magnitude of malnutrition in HIV infected children was high according to previous hospital based studies conducted in Ethiopia and India [11, 12].

On the other hand, malnutrition is one of the determinant factors for survival of HIV infected children [11, 13, 14]. Paton *et al*. demonstrated decreased survival of HIV infected patients if they had malnutrition at the time of initiation of ART [15]. Wasting and early growth faltering increased the risk of death among HIV infected children [16, 17]. Agarwal *et al*. reported the significant association between CD4 percentage values of HIV infected children and the presence of malnutrition [18]. Administering ART has been shown to be associated with significant increase in weight and height Z-scores after initiation of the drugs [19].

There is scarcity of data on immunological response following initiation of ART and its relationship with growth indicators. Understanding the pattern of nutritional and immunologic recovery following initiation of ART would be a potentially helpful information to optimize the follow up care in this specific patient population. In the current study we hypothesize that the change in anthropometric indices can be used as a proxy indicator of immunologic recovery with a potential use for follow up in resource limited settings to optimize or target the use of investigations of limited availability. We also hypothesize that the improvement in anthropometric indices is different with time on ART.

## Methods and Subjects

Data were collected from June 3, 2013 to September 20, 2013 among eligible HIV infected under-five children who were seen during their follow up at five public hospitals of Addis Ababa. The included hospitals were Tikur Anbessa Specialized Hospital, Yekatit12 Hospital, Saint Paul’s Hospital, Zewditu Memorial Hospital and ALERT Hospital. The hospitals are the largest in capacity in the capital Addis Ababa. All of them have established pediatric HIV clinics and trained staff.

From a computerized registration list, names and identification number of those children who were under-five and who were on first line ART was carefully selected. Samples of 202 children who were on first line ART and had at least one follow up visit were carefully selected and anonymized for the purposes of the current study.

A structured questionnaire was developed and used to collect data. Socio-demographic, anthropometric and clinical data were collected both at time of ART initiation and follow up. The questionnaire was pretested at a different hospital and translated to local language and also back translated to English. Data collection was done by nurses who had at least 2 years of work experience in the ART clinics. Skill assessment on anthropometric measurement and refresher hands-on training was given to the data collector nurses. They were also supervised and continuously supported by a pediatrician.

Socio-demographic characteristics were collected by interviewing the parent or guardian of the child. Collected socio-demographic information included age and sex of the child, family size, educational status of the mother, marital status of the mother, and frequency of feeding in the past twenty four hour period, and monthly income of the family.

Children are followed more frequently in the first three months of initiation of ART and then follow up becomes every three months unless there is a new illness or signs of drug side effects or evidences suggesting treatment failure are observed [20]. In the current study the minimum follow up while on ART was 6 months with median of 1.69 (IQR: 1.08–2.63) years. Anthropometric indices were cross-sectionally assessed using measurements at the time of enrollment to ART care (collected from patient charts) and also by directly measuring at the time of the survey. Follow up weight and height measurements were done when the child came to the hospital for his/her regular follow up. Weight was measured using weighing scale (Seca gmbh & co., Germany) which was calibrated twice daily. It was recorded to the nearest 0.1kg. For those less than two years, length was measured using a measuring board on a recumbent position. For those two years and above, height was measured on a standing position. Height/length was recorded to the nearest 0.1cm. MUAC was measured using the colored WHO MUAC tape.

Collected clinical data included age at HIV diagnosis, age at ART initiation, age at entry to follow up, WHO HIV/AIDS disease stage, adherence status to HAART, CD4 percentage, Cotrimoxazole prophylactic therapy, and previous history of hospital admission, These clinical data were regularly updated at each patient visit and recorded on the patient follow up chart and we took the most recent values (within six month preceding the survey).

A child was considered orphan if he/she had lost both of his/her parents. A child’s frequency of feeding was assessed based on the number of times the child fed over the previous 24 hours by the data collector nurses. The mother or caregiver was also asked if they have adequate supplies of food. Adherence to ARV drugs was assessed as poor/fair and good based on left over pill count and attendant’s report. Adherence is poor when the child takes less than 85% of the dose, fair when he/she takes 85–94% of the dose and good when he/she takes 95% and above of the dose.

Data were entered using EPI Info version 3.5.1 and then cleaned and checked for completeness. Then it was exported to and analyzed using STATA version 13.0 (StataCorp, Texas, USA) and ENA for SMART 2011 software. WAZ, HAZ and Weight-for-height Z-score (WHZ) were calculated using ENA for SMART 2011 software. The 2006 WHO reference standard was used to define malnutrition. Malnutrition was diagnosed when the anthropometric Z-score of the child falls -2SD below the median of the reference population.

Linear regression analyses was used to assess the relationship between CD4 percent and change in anthropometric Z-scores and predictors of anthropometric Z-scores improvement respectively. Bivariate and multiple linear regression were used to assess the predictors of anthropometric Z-score change. P-value less than 0.05 was taken as statistically significant.

## Ethical Consideration

Ethical approval was obtained from Research Ethics Committee of Addis Ababa University School of Public Health, the AHRI/ALERT Ethics Review Committee (AAERC), Addis Ababa Regional Health Bureau Research Ethics Committee, Saint Paul’s Hospital Millennium Medical College Institutional Review Board and from the Research Ethics Committee of the Department of Pediatrics and Child Health of Addis Ababa University. Verbal and written consent was obtained and documented from the caregivers. Confidentiality was assured by making the forms anonymous.

## Results

During the study period, we enrolled a total of 202 children who were started on first line ART and who came for follow up at five referral hospitals in Addis Ababa, Ethiopia. We included 46(22.8%) from Tikur Anbessa; 35(17.3%) from Yekatit 12 Memorial Hospital; 81(40.1%) from ALERT Hospital; 29(14.4%) from Zewditu Memorial Hospital and 11(5.5%) from St. Paul’s Hospital. The median age of the participants was 4.1 (Interquartile rage (IQR): 3.3–4.9) years. More than half (52.48%) were female. Seventeen (8.42%) are orphaned; for the majority (83.17%), their mothers are the caregivers. The median age at diagnosis of HIV was 1.4 (IQR: 0.6–2.8) years (Table 1).

**Table 1:** Demographic and clinical characteristics of under five children taking ART who had follow up in the five public hospitals in Addis Ababa, Sep. 2013 (n = 202).

Variable	Number	Percent
**Age**		
Younger than 2 years	16	7.9
2 to 5 years	186	92.1
**Sex**		
Male	106	52.5
Female	96	47.5
**Feeding frequency in the past 24 hours**		
1–3	89	44.1
3–6	103	51
>6	10	4.9
**Description of food eaten in the child’s home in the past 12 months**		
Enough food in kind and amount	22	10.9
Enough food but not in variety	43	21.3
Sometimes no enough food for the family	24	11.9
Always no enough food for the family	113	55.9
**Hospital admission in the past 6month**		
No	170	84.2
Yes	32	15.8
**WHO clinical stage of HIV/AIDS**		
I	24	11.9
II	84	41.6
III	79	39.1
IV	15	7.4
**Is child taking Cotrimoxazole**		
No	8	4
Yes	194	96
**CD4 percentage value**		
<25	121	60.2
25–35	38	18.9
>35	42	20.9
**Age at diagnosis of HIV**		
<18 months	110	54.5
≥18 months	92	45.5
**Getting nutritional supplement**		
No	126	62.4
Yes	76	37.6
**Age at ART initiation (n = 161)**		
<18 months	75	46.6
≥18 months	86	53.4
**ART adherence status**		
Poor/fair	9	4.5
Good	190	94.1
Missing	3	0.5
**Duration of ART intake**		
<1 year	73	36.7
1–2 year	61	30.7
>2 years	65	32.7

The median age of ART initiation and entry to follow up care were 18(IQR: 9.75, 31) and 16(IQR: 6, 32) months respectively. Thirty six (18.1%) children had diarrhea which lasted for more than two weeks before the follow up time. History of hospital admission over the past six months before the time of data collection was reported by 45 (22.6%) children. Ninety four (46.5%) had developed WHO HIV/AIDS clinical stage3 or 4 defining illness. One hundred eighty nine children (95.0%) were taking cotrimoxazole prophylactic therapy.

**Fig 1:**
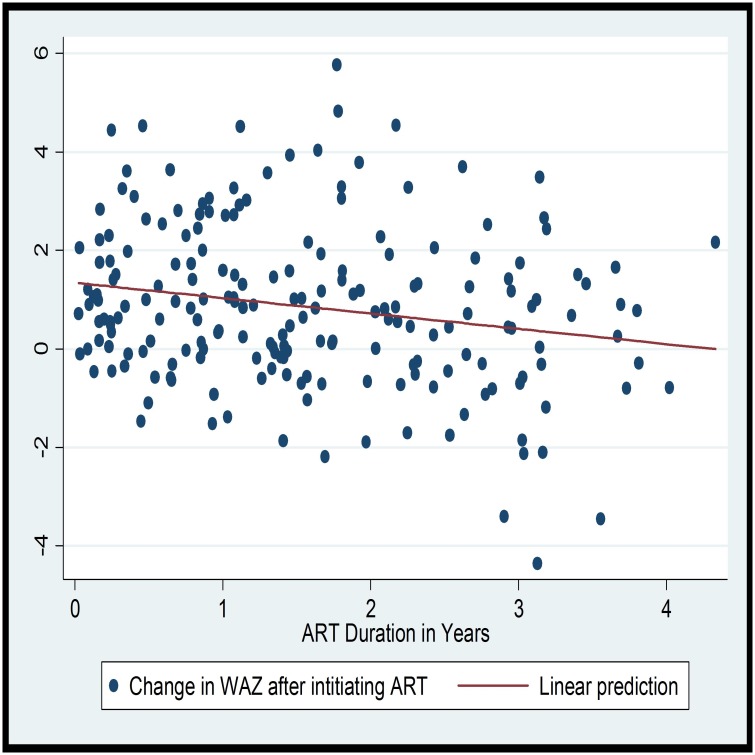
Scatter plot and linear prediction of change in WAZ and duration of ART. Each point in the scatter plot corresponds to the change in WAZ.

**Fig 2:**
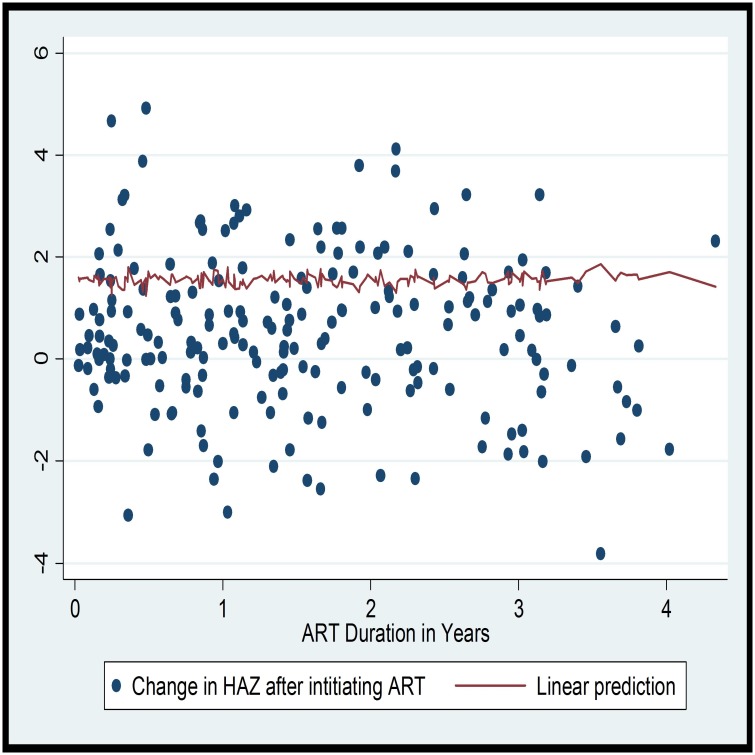
Scatter plot and linear prediction of change in HAZ and duration of ART. Each point in the scatter plot corresponds to the change in HAZ.

The magnitude of underweight, stunting and wasting—as defined by less than -2 ZS—at the time of ART initiation were 39.5%, 71.3% and 16.3% respectively. Whereas, at the end of follow up, 16.5%, 62.9% and 1.0% had underweight, stunting and wasting respectively (p-value <0.0001). There was a significant improvement in all the anthropometric indices at any follow up visit. The most significant change was for weight for age (WAZ) (t = 5.802, p-value<0.0001) (Table 2). While looking at the pattern of improvement with time on follow up, it was observed that there is an inverse linear relationship between improvement in WAZ and duration of ART (R^2^ = 0.04; F (1, 158); p = 0.013). On the linear prediction, it can be seen that the improvement in WAZ is high during the first few years of follow up (Fig 1). However, no linear relationship was observed between duration of ART and HAZ or WAZ (Figs 1 and 2).

**Table 2:** Nutritional status of the children at the time of ART initiation and during the data collection time, Sep 2013, Addis Ababa (n = 202).

Anthropometric index	Pre-ART Mean(95%CI)	At any follow up (6–53 months) Mean(95%CI)	Difference Mean(95% CI)	T	p-value
Weight for age Z Score	-1.77(-2.03 to -1.53)	-1.01(-1.18 to -0.84)	0.77(0.51 to 1.03)	5.802	< 0.0001
Height for age Z Score	-2.97(-3.22 to -2.73)	-2.55(-2.71 to -2.38)	0.427(0.19 to 0.66)	3.603	0.0004
Weight for height Z Score	-0.10(-0.43 to 0.23)	0.71(0.50 to 0.92)	0.81(0.47 to 1.15)	4.704	<0.0001
CD4 percentage	19.78(16.23 to 23.34)	26.99(25.66 to 28.32)	10.95(6.14 to 15.76)	-4.357	<0.0001

**Fig 3:**
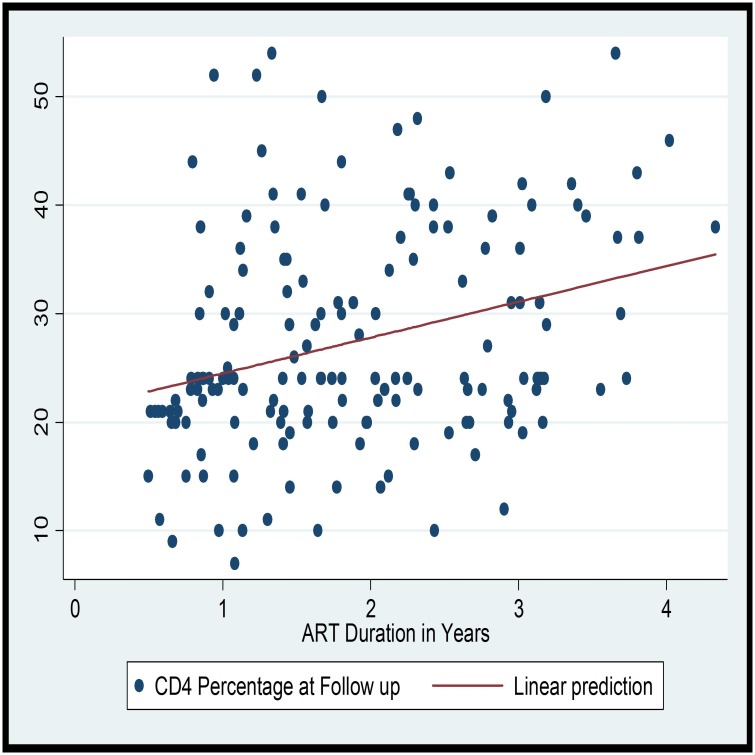
Scatter plot and linear prediction of CD4 Percentage at Follow up and Duration of ART.

**Fig 4:**
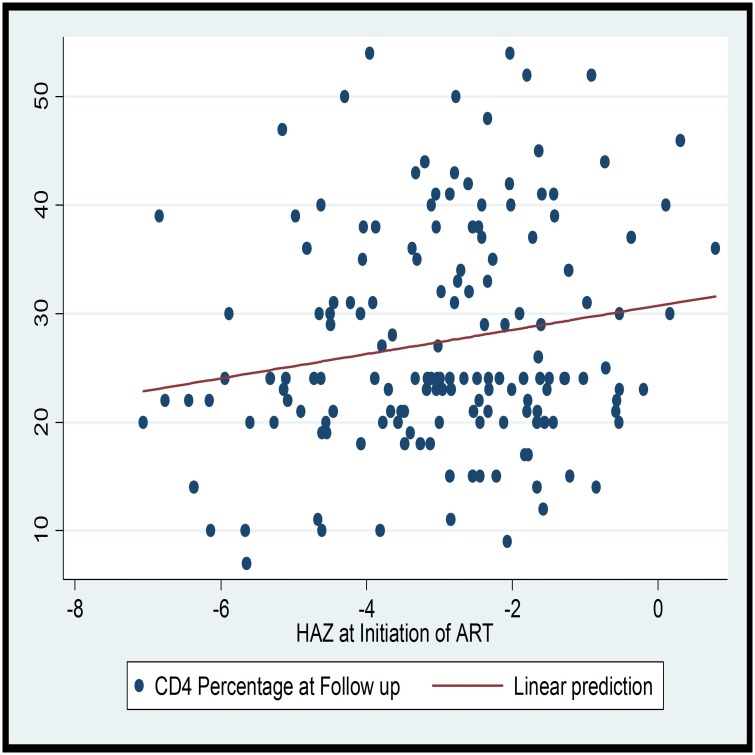
Scatter plot and linear prediction of HAZ at ART initiation and CD4 at Follow up.

The median duration of follow up while on ART was 1.69 (IQR: 1.08–2.63) years. The mean CD4 percentage at follow up was 26.99% (SD = 9.58) with 39.8% having a CD4 of 25% or above. The CD4 percent at follow up and duration of ART are linearly correlated (Coef = 0.009; F (1, 158); R^2^ = 0.09; p-value = 0.000). On the linear prediction, it can be observed that the CD4 percentage increases with the duration of ART in the first four years. On assessing if anthropometric indices correlate with immunologic recovery, there was a statistically significant linear relationship between both HAZ at ART initiation (Coef. = 1.11; R^2^ = 0.03; p-value = 0.029) and HAZ at follow up (Coef. = 1.92; R^2^ = 0.05; p-value = 0.002) with the CD4 percentage measured at least six months after initiation of ART (Table 3). However, the WAZ and WHZ were not associated with the CD4 percentage measured at least six months after initiation of ART (Figs 3 and 4).

**Table 3:** Relationship between anthropometric indices measured at ART initiation and at follow up (after at least 6 months of ART initiation) and percent CD4 count at Follow up.

Anthropometric Indices	CD4 Percentage at Follow up			Change in CD4 Percentage		
	Coef.	R2	p-value	Coef.	R2	p-value
**At ART initiation**						
Weight-for Age ZS$	0.89	0.02	0.074	-0.003	0	0.863
Height-for-Age ZS	1.11	0.03	0.029	-0.024	0.059	0.107
Weight-for-Height ZS	0.14	0	0.722	-0.003	0	0.887
**At any duration of follow up during data collection**						
Weight-for Age ZS	0.05	0	0.933	-0.017	0.067	0.086
Height-for-Age ZS	1.92	0.05	0.002	0.016	0.068	0.083
Weight-for-Height ZS	0.87	0.01	0.091	-0.037	0.203	0.001
**Change during follow up**						
Change Weight-for Age ZS	-0.89	0.02	0.067	-0.014	0.021	0.388
Change Height-for-Age ZS	-0.31	0	0.566	0.03	0.087	0.073
Change Weight-for-Height ZS	-0.47	0.01	0.211	-0.033	0.067	0.116

^$^Z-score

The mean time on follow up was longer for children who have not gained or have lost WAZ at follow up as compared to those who have gained WAZ (mean (SD): 523.1(32.1) vs 658.0(50.7); p-value = 0.025). On bivariate linear regression, duration of ART (*r*^*2*^ = 0.038, p-value = 0.013) and WHO clinical stage (*r*^*2*^ = 0.034, p-value = 0.020) predicted change in WAZ at any follow up while age at diagnosis of HIV (*r*^*2*^ = 0.040, p-value = 0.012), age at initiation of ART(*r*^*2*^ = 0.065, p-value = 0.000), availability of food (*r*^*2*^ = 0.035, p-value = 0.018) and taking cotrimoxazole preventive therapy (*r*^*2*^ = 0.020, p-value = 0.018) predicted the change in HAZ at any follow up. Adherence which was reported to be good in the majority 190 (94.1%) of the children was not significantly associated with change in WAZ at follow up. (Table 4). On multiple linear regression, duration on ART (*Std*. *Err*. = 0.206, *t* = -1.99, p-value = 0.049) and level of maternal education (*Std*. *Err*. = 0.290, *t* = 2.64, p-value = 0.009) were the only independent predictors of the change in WAZ and change in HAZ at any follow up visit respectively (Table 5).

**Table 4:** Bivariate correlation between independent variables and the change in weight-for-age (WAZ), height-for-age (HAZ) and weight-for-height (WHZ) Z-scores at any follow up among preschool children attending ART clinics of 5 hospitals.

Variable	HAZ Difference		WAZ Difference		WHZ Difference	
	*r*^*2*^	p-value	*r*^*2*^	p-value	*r*^*2*^	p-value
Age at HIV Diagnosis (years)	0.040	0.012	0.009	0.235	0.004	0.417
Age at ART initiation (years)	0.065	0.000	0.022	0.062	0.012	0.174
Duration of ART (years)	0.003	0.517	0.038	0.013	0.019	0.082
Sex	0.000	0.912	0.006	0.234	0.010	0.215
CD4 count at follow up	0.020	0.552	0.021	0.067	0.010	0.211
Change in CD4 percent	0.087	0.072	0.021	0.388	0.067	0.116
Food availability	0.035	0.018	0.019	0.082	0.002	0.605
Food supplements	0.007	0.291	0.001	0.692	0.014	0.140
Adherence to ART	0.000	0.800	0.003	0.477	0.010	0.215
Maternal Age	0.005	0.327	0.011	0.231	0.011	0.227
Mother’s education	0.053	0.009	0.024	0.071	0.000	0.994
Mother alive	0.000	0.853	0.017	0.090	0.020	0.072
Marital status	0.003	0.509	0.005	0.367	0.006	0.324
Birth interval	0.038	0.109	0.003	0.680	0.000	0.930
Co-trimoxazole intake	0.020	0.018	0.011	0.195	0.004	0.405
WHO stage (pre-ART)	0.020	0.080	0.034	0.020	0.024	0.049
Diarrheal diseases	0.003	0.488	0.002	0.542	0.002	0.584

**Table 5:** Results of multiple linear regression analysis of changes in Z-scores of height-for–age (HAZ), weight-for-age (WAZ) and weight-for-height (WHZ) among preschool children attending ART clinics of 5 hospitals.

Variable	HAZ Difference^†^			WAZ Difference^‡^			WHZ Difference^¶^		
	*Std Err*.	*t*	p-value	*Std Err*.	*t*	p-value	*Std Err*.	*t*	p-value
Age at HIV Diagnosis (years)	.019	0.17	0.863						
Age at ART initiation (years)	.017	1.64	0.103	.012	1.23	0.221			
Duration of ART (years)	.179	1.16	0.247	.206	-1.99	0.049	.223	-1.70	0.091
CD4 count at follow up	.012	-0.72	0.471	.014	1.17	0.243			
Food availability	.123	-1.25	0.215	.142	-0.95	0.344			
Mother’s education	.290	2.64	0.009	.337	1.19	0.238			
Co-trimoxazole intake	.615	-1.66	0.098						
WHO stage (pre-ART)	.170	0.59	0.554	.196	1.01	0.314	.221	1.84	0.067
_constant	1.04	0.24	0.809	.952	0.44	0.661	.883	1.50	0.136

^†^Adjusted *r*^*2*^ = 0.113, *p-value* = 0.003;

^‡^Adjusted *r*^*2*^ = 0.098, *p-value* = 0.038;

^¶^ Adjusted *r*^*2*^ = 0.113, *p-value* = 0.031

## Discussion

The current study assessed the improvement in anthropometric Z-scores following initiation of first-line ART to perinatally HIV infected under-five children at five public hospitals in Addis Ababa, Ethiopia. A decline in the prevalence of malnutrition was observed following initiation of ART. The proportion of malnutrition at ART initiation as compared to the proportion at any follow up visit was underweight; 39.5% vs16.5%, stunting; 71.3% vs 62.9%, and wasting; 16.3%vs 1.0%. ART is associated with rapid recovery from acute malnutrition demonstrated by significant decline in proportion of children who would be classified as wasted. And, gradual decline in stunting as evidenced by an improving HAZ was observed. The improvement in WAZ was found to be inversely related to the duration of ART. The longer the time on ART, the smaller the WAZ gains after ART initiation. Duration on ART at the time of follow up was an independent predictor of improvement in the WAZ in under-five children. HAZ was found to have a linear relationship with the CD4 percentage measured at least six months after initiation of first line ART.

It was observed that there is a significant improvement in WAZ during the first years of follow up after ART initiation. However, as the duration of ART intake increases, the WAZ gain was smaller possibly indicating the loss of the beneficial effect of ART with time and possibly due to virological treatment failure. It could also be due to the leveling off of WAZ improvement once a certain WHZ is achieved or due to the normal growth deceleration of the cohort. Though not assessed in the current study, type of ART regimen and drug side effects could be possible contributors to the change in WAZ. Moreover, ART may not be the only factor affecting the nutritional improvement; it could also show the socioeconomic consequences of HIV infection with time or macro and micro nutrient deficiencies in the affected children. Researches done among children and adults on first-line ART have similarly demonstrated the positive effect of ART initiation on improvement of nutritional status [19, 21]. The anthropometric improvement could be attributed to improving appetite and better intake, lower metabolic requirement because of less opportunistic infections. In our study, CD4 percentage above 35% at follow up time was the only independent predictor of improvement in WAZ after ART initiation. A similar finding was reported by a study done in India where CD4 percentage was found to correlate significantly with presence of protein energy malnutrition in HIV infected children [18]. Children who were initiated on first line ART at a higher CD4 count were observed to have a better improvement and lower treatment failure [22–24].

Congruent with other studies which assessed immunologic recovery in children [25–27], our findings also show that there is a positive relation between anthropometric indices and immunologic recovery at follow up. Specifically, the HAZ at both ART initiation and follow up was found to be positively correlated with the CD4 percentage at follow up. Similar reports have been published thus far for adults and older children [25, 28, 29].

In the current study, the presence of diarrhea, and taking cotrimoxazole prophylactic therapy did not show statistically significant associations with change in WAZ following initiation of ART. This could be because of the small number of children who were reported to have the conditions and most children had already been started on cotrimoxazole prophylactic therapy. However, several other studies have reported that these variables are associated with malnutrition in HIV infected patients [22, 26, 30]. Cotrimoxazole prophylactic therapy has been shown to improve their survival in children [31]; and improvement in anthropometric Z-scores among adults [32].

The current study addressed important points including relationship between duration on follow up while on ART, anthropometric improvement and immunologic recovery. However, there were several limitations to the study. In the current study, we used growth measurements at only two points which is less informative than a more frequent measurement and growth monitoring using regular plots and growth follow up through monitoring growth velocity. Additionally, since the follow up time for a third of the children was less than one year, it is difficult to observe a change in height/length. Moreover, since pre-ART anthropometric values were taken from records, we cannot be certain about their accuracy; and because of the presence of missing records especially the percent CD4 during pre-ART, there were fewer data points for the change in CD4 percentage. While food inadequacy could be one of the main causes of undernutrition, comprehensive checklists to assess the adequacy of feeding couldn’t be done in the current study.

In conclusion, there was a significant improvement in all anthropometric indices at any follow-up after initiation of first-line ART among under-five children. The WAZ improvement trend was inversely related to the duration of ART. HAZ was positively correlated with immunologic recovery on follow up. The findings indicate that anthropometric indices could be taken as proxy indicators of immunologic recovery for under-five children who were initiated on first line ART. Paying a closer attention to the anthropometric indices could be an addition to the virologic and immunologic follow up of under-five children on first-line ART or an alternative follow up tool where resources are not available.
